# Vital pulp therapies in permanent teeth: what, when, where, who, why and how?

**DOI:** 10.1038/s41415-025-8560-3

**Published:** 2025-04-11

**Authors:** Thibault N. E. Colloc, Phillip L. Tomson

**Affiliations:** 695725814578434689702 Clinical Lecturer and Honorary Specialty Trainee in Endodontology, University of Dundee, United Kingdom; 149394846267159633278 Senior Clinical Lecturer and Honorary Consultant in Restorative Dentistry, University of Birmingham, United Kingdom

## Abstract

The dentine-pulp complex is a unique organ that provides nourishment to the dentine and supports the tooth's innervation and defence system. Preserving its health is a key aspect of minimally invasive dentistry. Vital pulp therapies (VPTs), as defined by the European Society of Endodontology, are ‘treatment strategies aimed at maintaining the health of all or part of the pulp'. These therapies are particularly useful when pulp inflammation, caused by caries or trauma, threatens pulp vitality and may otherwise lead to necrosis and the need for invasive treatment. VPTs offer several advantages, including the retention of the pulp's immunological functions, maintenance of the structural integrity of the tooth, reduced patient pain and simplified treatment protocols. Additionally, these therapies lower the costs and inconvenience associated with more invasive treatments, benefiting both patients and society. A range of protocols and key factors promoting successful outcomes have been described to guide dental practitioners in adopting VPTs where appropriate. Future considerations for pragmatic research and implementation in primary care are essential to create a sustainable use of VPTs, thus promoting better oral health.

## Key points

- Provides an updated review of the evidence for using vital pulp therapies in permanent teeth and the guidelines available.
- Suggests a step-by-step clinical guide for vital pulp therapies.
- Advises on the materials available for vital pulp therapies.
- Discusses how to address future considerations and limitations.

## Introduction

The concept of minimally invasive dentistry is well-established and can be described as ‘a systematic respect for maintaining original tissue'.^1^ Preserving permanent teeth is essential for patients to maintain good health and function in their daily life, for communicating, eating, or for social purposes.^2^

The dentine-pulp complex is a unique connective tissue that consists of hard dentine, which forms most of the tooth structure, and the dental pulp, which provides nutrition to the dentine and supports the tooth's innervation/defence system.^3^ This ‘complex' can respond to any insult, such as caries or trauma, by a combination of an inflammatory response and promotion of mineralisation to preserve/maintain itself.^4^ Bacteria, the main cause of pulpal disease,^5^ may invade the dentine-pulp complex via many different routes, such as carious lesions, traumatic loss of tooth tissue, cracks, or defective restoration margins. This bacterial infection can lead to pulpal inflammation that may be managed by successful defensive response or an iatrogenic intervention. If it is not, it may enter a stage of ‘necrobiosis', where the pulp degenerates and progressively necroses until the entire root canal system becomes pulpless and infected. At any point during this process, simultaneously, the periapical tissues may become inflamed, resulting in breakdown of these tissues also. Once pulp inflammation is established or indeed if it becomes necrotic in restorable teeth, root canal treatment (RCT) is widely considered the only option to preserve and restore the tooth to healthy function. However, if the disease is intercepted at the appropriate stage, alternative, less invasive, biologically based therapies are available to maintain pulp health and prevent periapical pathology: these are called vital pulp therapies (VPTs).

## What are vital pulp therapies?

VPTs or vital pulp treatments are defined by the European Society of Endodontology (ESE) as ‘treatment strategies aimed at maintaining the health of all or part of the pulp'.^6^ Different VPT approaches are indicated depending on the severity and progress of disease and are outlined in Table 1.^7^

**Table 1:** Description of vital pulp therapies^6^^,^^7^ (diagrams reproduced with permission from the British Endodontic Society)

**Procedure**	**Definition**	**Indication**	**Materials used**	**Diagrams**
Indirect pulp capping	Application of a biomaterial onto a thin dentine barrier in a one-stage complete carious-tissue removal technique generally to hard dentine	Asymptomatic carious lesion without pulp exposure where selective caries removal is not indicated Reversible pulpitis without pulp exposure	Aseptic field using dental dam with magnification and disinfection protocol Hydraulic calcium silicate cement or glass ionomer cement followed by restorative material as required according to clinical presentation	**Figure:** 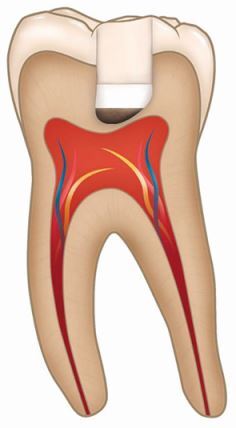
Direct pulp capping	Application of a biomaterial directly onto the exposed pulp, before immediate placement of a permanent restoration	Asymptomatic carious lesion with pulp exposure Reversible pulpitis with pulp exposure	Aseptic field using dental dam with magnification and disinfection protocol Hydraulic calcium silicate cement followed by restorative material as required according to clinical presentation	**Figure:** 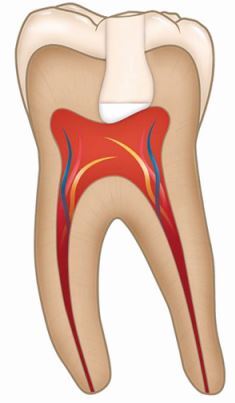
Pulpotomy (partial or full)	Removal of part or the full extent of coronal pulp tissue after exposure, followed by application of a biomaterial directly onto the remaining pulp tissue before placement of a permanent restoration	Asymptomatic carious lesion with pulp exposure Reversible pulpitis with pulp exposure Irreversible pulpitis	Aseptic field using dental dam with magnification and disinfection protocol Hydraulic calcium silicate cement followed by restorative material as required according to clinical presentation	**Figure:** 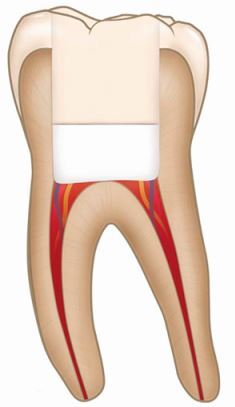
Pulpectomy	Total removal of the pulp from the root canal system followed by root canal treatment, before placement of a permanent restoration	Irreversible pulpitis Pulp necrosis	Aseptic field using dental dam with magnification and enhanced disinfection protocol Gutta-percha with a root canal sealer (epoxy resin; zoe or calcium silicate) followed by restorative material as required according to clinical presentation	**Figure:** 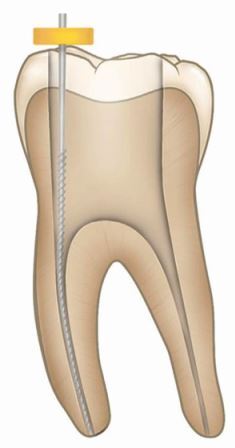

## When and where are vital pulp therapies indicated?

Carious lesions are a common cause of an insult to the dentine-pulp complex, which may lead to pulpal disease and potentially pulp necrosis if left untreated. Early management of dental caries, respecting the effect such interventions may have on the dental pulp, may prevent the need for any endodontic intervention. The restorative cycle is initiated as soon as a restoration is placed in a tooth and will inevitably have an impact on the dentine-pulp complex, subsequently leading to more invasive treatments over time.^8^ VPTs provide an additional option in the treatment armamentarium in the management of the diseased pulp to prevent or delay the need for RCT and preserves the immunological functions of the tooth.^9^ Different clinical presentations and diagnoses in an elective or emergency setting which may lead to performing VPT are as follows.

### Normal pulp/symptomatic pulpitis without carious lesion or pulp exposure

This may arise when replacing an existing deep restoration, managing a crack, or following a recent traumatic dental injury. In a caries-free cavity with a thin dentinal wall protecting the pulp from exposure, indirect pulp capping with therapeutic material, such as hydraulic calcium silicate cement (HCSC), may be necessary to maintain pulp health. This procedure should be performed using aseptic techniques, including isolation with a dental dam and cavity disinfection with sodium hypochlorite (NaOCl), before restoring the tooth with an appropriate direct and/or indirect restoration, depending on the remaining tooth structure.^7^^,^^10^

### Normal pulp/symptomatic pulpitis with very deep carious lesion

**Fig. 1:**
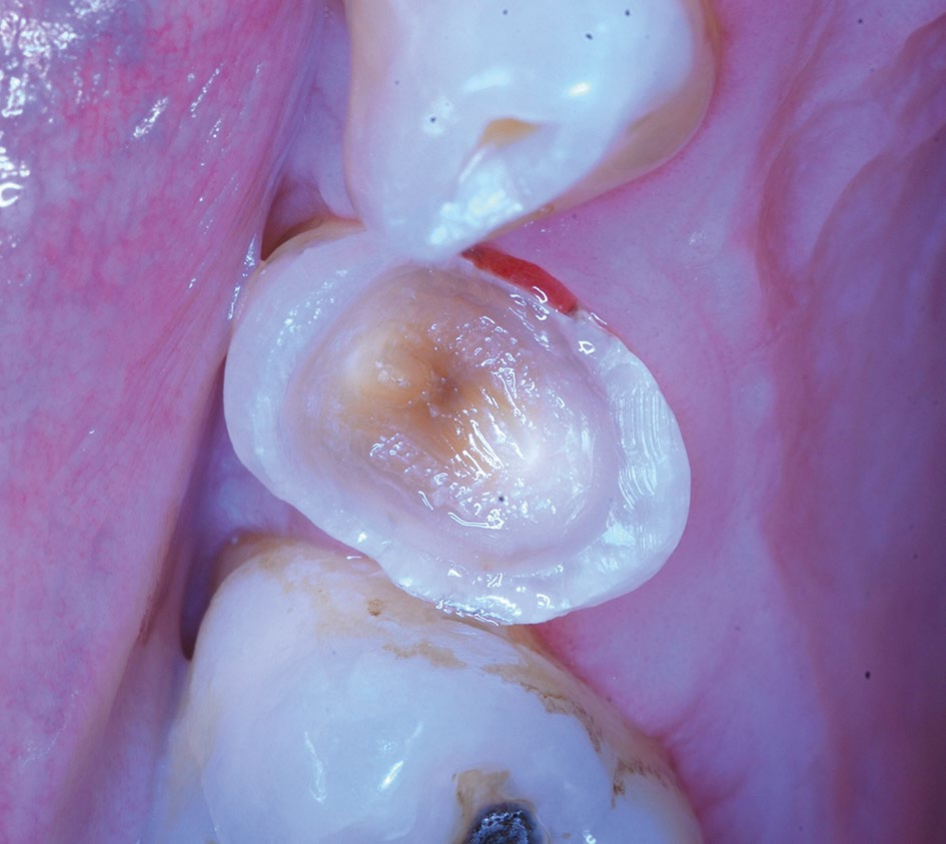
Completed cavity preparation following treatment of caries using selective caries removal strategy in upper right first premolar showing clean amelo-dentinal junction and firm caries overlying pulp

In this scenario, the patient may not report any symptoms from the tooth associated with the carious lesion. After carefully confirming the diagnosis through clinical and radiographic investigations, an initial strategy of selective caries removal is adopted, with the first operative step being clearing the amelo-dentinal junction (caries-free hard dentine at the periphery of the cavity). Aseptic field using dental dam with magnification and disinfection protocol should be used. Depending on the depth of the lesion, two endpoints may be considered for selective excavation. Selective caries removal to firm dentine (should be resistant to excavation using hand instruments)^6^ is often the treatment of choice for moderately deep lesions where the ‘leathery' dentine is left behind pulpally (Fig. 1). In the case of deep cavitated lesions extending into the pulpal third or quarter of dentine, selective removal to soft dentine (can be excavated with minimum resistance using hand instruments)^6^ should be applied, where soft carious tissue is left over the pulp to avoid exposure and ‘stress' to the pulp, encouraging pulp health, while peripheral enamel and dentine are prepared to hard dentine (should be sound and resistant to probe penetration and scratching). Such an approach has been shown to reduce the risk of pulp exposure.^11^ However, if the carious lesion is too deep, pulp exposure may be unavoidable. In such cases, direct pulp capping (Class II or carious) or some form of pulpotomy (depending on the status of the pulp and the ability to control haemorrhage) may offer a more predictable and successful outcome. In this case, it is assumed that the bacterial load is much greater and the approach is more radical; if pulp exposure occurs, complete caries removal should be performed using an aseptic technique to reduce the bacterial load before proceeding with VPT.^12^ The tooth is then restored with an appropriate direct and/or indirect restoration, depending on the remaining tooth structure.

### Normal pulp and accidental pulp exposure

**Fig. 2:**
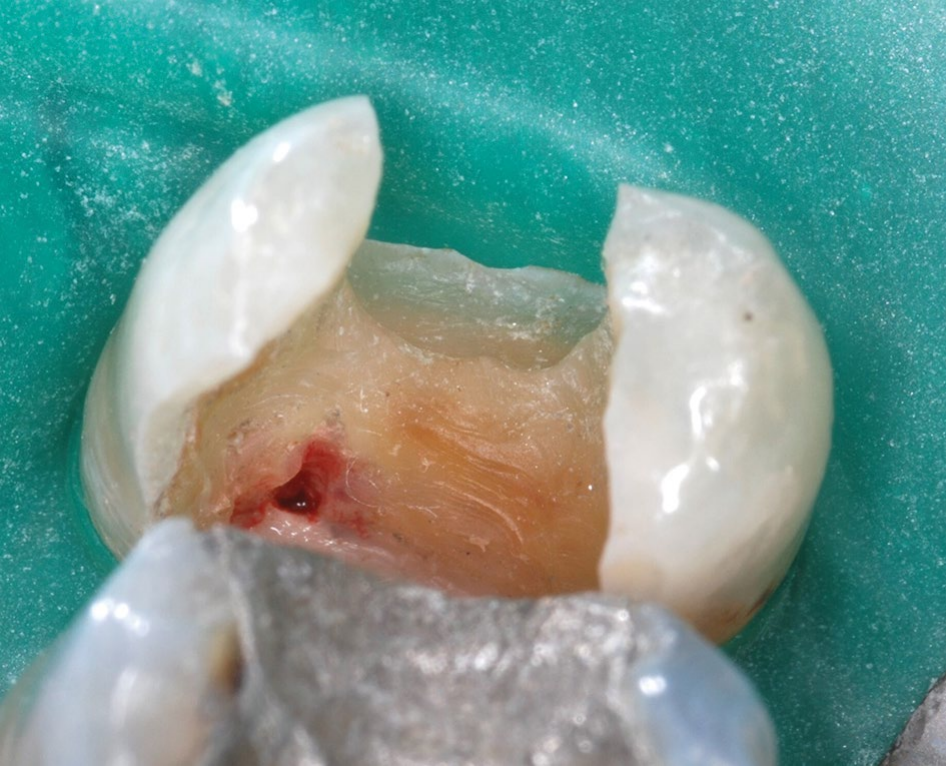
Unexpected pulp exposure of pulp horn during replacement of restoration in upper right second premolar tooth

This scenario may arise when replacing a deep restoration, managing a crack, or following a traumatic dental injury. In such cases, the pulp exposure is surrounded by sound dentine and bacterial contamination is less likely (Fig. 2). To promote pulp vitality and healing, it is recommended to use an aseptic technique using dental dam with magnification and disinfection protocol for direct pulp capping (Class I or non-carious) before restoring the tooth with an appropriate direct and/or indirect restoration, depending on the remaining tooth structure.^7^

### Symptomatic irreversible pulpitis with normal apical tissues/periodontal ligament widening/apical tissue breakdown

**Fig. 3:**
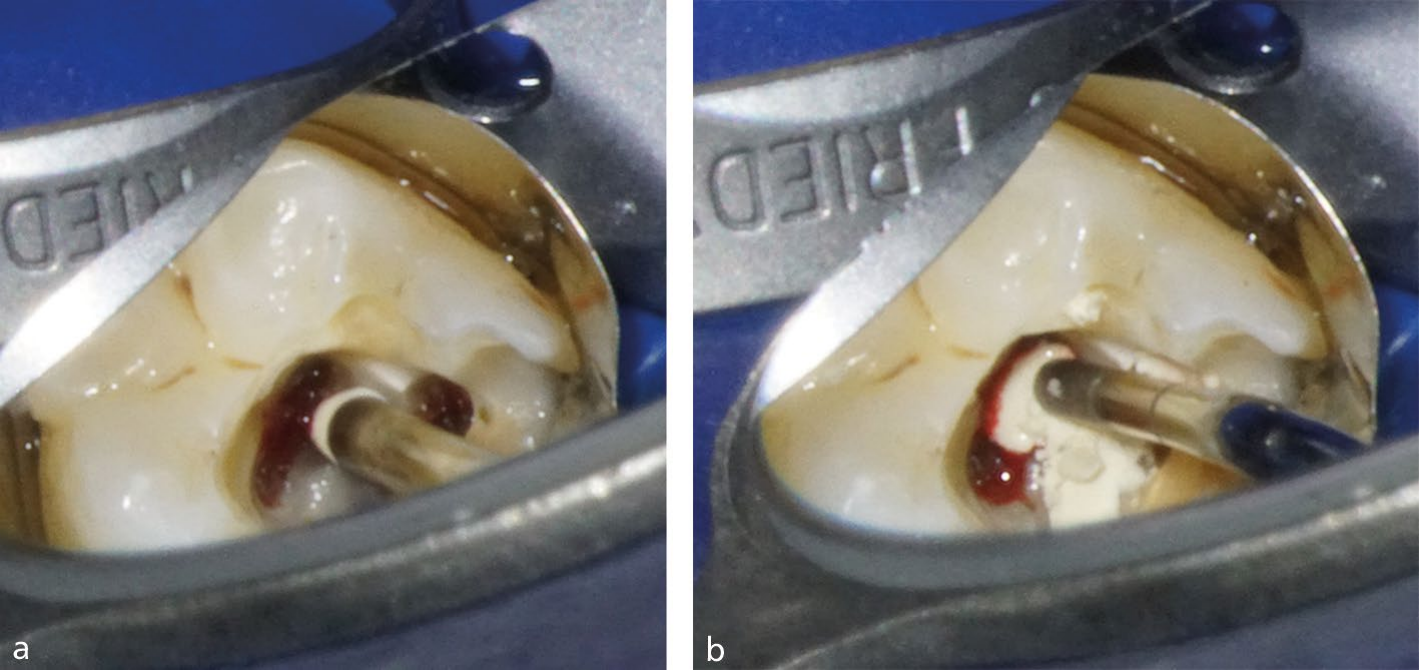
a) Biodentine expelled from carrier instrument directly on pulp floor and viable pulp tissue at orifice of distal root canal following full pulpotomy in lower right second molar. b) Manipulation of Biodentine using Glick 1 hand instrument on pulp tissue and pulp floor in panel a

The management of symptomatic irreversible pulpitis is a common emergency encountered in dental practice. Success in treatment depends heavily on an accurate diagnosis, both clinically and radiographically. After achieving effective anaesthesia and removing all caries and defective restorations, a partial or full pulpotomy may be a successful treatment option for preserving the vitality of the radicular pulp, provided it is performed under strict aseptic conditions with dental dam, disinfection protocol and using magnification, ideally.^13^ Clinical observation of haemorrhage is used as a surrogate marker for the degree of inflammation of the pulp. Haemostasis must be achieved following either a partial or full pulpotomy. It is preferable to place a HCSC in direct contact with the pulp tissues. A HCSC should be placed in direct contact with the pulp tissues before restoring the tooth with an appropriate direct and/or indirect restoration, depending on the remaining tooth structure (Fig. 3). Alternatively, if pulp necrosis is observed (whether partial or complete) or if haemostasis cannot be achieved, a pulpectomy and RCT should be considered instead.^13^

### Necrotic pulp with normal apical tissues/apical periodontitis

When pulp necrosis is confirmed clinically and radiographically, and the tooth is deemed restorable, RCT should be performed as soon as possible to prevent the risk of acute infection. These procedures must be conducted under strict aseptic conditions, including aseptic field with dental dam, magnification and disinfection protocols. After completing the treatment, the tooth should be restored with an appropriate direct or indirect restoration, depending on the remaining tooth structure following basic restorative principles.^14^^,^^15^^,^^16^

Although robust evidence is increasingly available to support clinical decision-making, areas such as diagnostic tests for determining pulp status remain contested, with a lack of conclusive evidence to fully support current practices.^17^^,^^18^^,^^19^ The newly developed ESE S3 guidelines provide a comprehensive summary of current evidence to support clinicians in the diagnosis and management of pulpitis, offering a clear framework for informed decision-making in VPT (Table 2). The principal aim of these guidelines is to provide expert- and evidence-based recommendations that reflect the most current and scientifically backed therapeutic strategies. By doing so, they not only inform clinicians, but also underscore areas of uncertainty where future research is essential. These recommendations help clinicians navigate through the complexity of diagnosing and treating vital pulp, especially in cases of reversible and irreversible pulpitis.^7^

**Table 2:** Summary table relating to vital pulp therapies^7^

**Question**	**Context**	**Management options (grade of recommendation)**			**Evidence**	**Consensus**
Effectiveness of vital pulp treatment in managing non- traumatic pulpitis associated with no or nonspontaneous pain	Patients with non-traumatic pulpitis associated with no or non-spontaneous pain in immature and mature permanent teeth	Selective/stepwise caries removal without pulp exposure	If pulp exposure direct pulp capping (open)*	If pulp exposure, pulpotomy (partial/full) (open)*	Expert-based evidence Position statement ESE (2019)	Strong
			Enhanced protocol (ie dental dam, antimicrobial lavage, magnification and use of a hydraulic calcium silicate cement)		Expert-based evidence Position statement ESE (2019)	Strong
	Patients with non-traumatic pulpitis associated with no or non-spontaneous pain and pulp exposure in mature permanent teeth	Direct pulp capping (open)	Pulpotomy (partial/full) (open)*	Pulpectomy (open)*	Supporting literature (Jakovljevic *et al.*, 2022)	Consensus
		Enhanced protocol (ie dental dam, antimicrobial lavage, magnification and use of a hydraulic calcium silicate cement)				
Effectiveness of pulpotomy compared with root canal treatment in managing nontraumatic pulpitis associated with spontaneous pain	Patients diagnosed with non-traumatic pulpitis associated with spontaneous pain in permanent teeth	Root canal treatment and restoration (weak)**	Pulpotomy and restoration (weak)**		Supporting literature (Tomson *et al.*, 2022)	Consensus
Effectiveness of root canal treatment for vital pulps compared with necrotic pulps in the presence or absence of signs of periradicular pathosis	Teeth with nonvital pulps	Root canal treatment and restoration (weak)**			Supporting literature (Rossi-Fedele & Ng, 2022)	Strong
Key: * = no significant evidence available, open to clinician's interpretation and experience based on the clinical scenario ** = low-level evidence available						

## Who benefits from vital pulp therapies and why?

Preserving pulp vitality and promoting minimally invasive endodontic treatment can present with multiple benefits to patients but also more broadly for dental public health purposes, as described by Wolters *et al*.^9^

### Preservation of immunological functions and retaining structural integrity of the tooth

Maintaining the viability of the dental pulp allows the induction of a biological response which will prevent the occurrence of pulp necrosis/apical periodontitis, thus preserving the tooth's defence system and delaying the need for subsequent endodontic treatment. This is particularly relevant in young permanent teeth with open apices, where preserving the pulp can ensure continued root development.^20^ There is also a correlation between the amount of residual natural tissue of a tooth and its long-term survival, which suggests minimally invasive procedures such as VPT will increase the survival of restored teeth.^16^^,^^21^

### Simplifying treatment procedures and avoiding treatment complications associated with difficult root canal anatomy

RCT in posterior teeth comes with high demands for a successful outcome. Deconstructing the treatment into procedural components highlights the complexity of it and the necessary steps required to achieve a satisfactory outcome. The procedural components might include placement of dental dam, access preparation, canal identification, canal negotiation, length determination, canal enlargement parameters, lavage and disinfection of the canal system (conventionally called irrigation and medication) and lastly, obturation.^22^ Complications can occur during each of these steps, leading to challenges which may render the tooth unrestorable or highly compromise their long-term survival (iatrogenic perforation, absence of patency, extrusion of root canal filling).^23^ Simplifying the treatment procedure using VPTs may delay the need for RCT and therefore increase the survival of the tooth by delaying the restorative cycle.^8^^,^^24^

### Suggested procedures cause little pain

Multiple studies have investigated the pain level of patients who have been treated with RCT compared to VPT.^13^^,^^25^Data suggest that full pulpotomy may have a lower incidence of postoperative pain compared to single-visit RCT.^25^ However, for the management of irreversible pulpitis, no significant difference was found for postoperative pain after seven days. Clinical success was high at one year (98% for both interventions) but decreased over time to 78.1% (pulpotomy) and 75.3% (RCT) at five years.^13^

### Reducing cost and inconvenience for patients and society

Cost-effectiveness and tooth survival are important considerations for both patients and healthcare professionals, as well as from a dental public health perspective, especially for government-funded healthcare systems. According to Schwendicke et al., direct pulp capping or pulpotomy is generally more cost-effective than RCT when managing pulp exposures. However, pulpotomy was found to be less costly than RCT.^26^^,^^27^A recent cost-effectiveness study focusing on the management of irreversible pulpitis in permanent teeth based on a United States remuneration model revealed that both pulpotomy and RCT were cost-effective at managing irreversible pulpitis in mature permanent teeth. RCT provided an additional health benefit (tooth retained 1.08 years longer with RCT) over a period of an individual's lifetime at an increased willingness-to-pay level, whereas pulpotomy was cost-effective at a lower willingness-to-pay level over an individual's lifetime.^28^

Climate change has led to an increasing awareness for the need to implement sustainability in all aspects of society, including healthcare and dentistry.^29^ Most healthcare systems aren't currently sustainable due to the high amounts of carbon dioxide (CO_2_) and waste generation.^29^ A recent study from Duane et al. assessed the environmental impact of RCT procedures, revealing that each procedure contributes 4.9 kg of CO_2_ emissions, or the equivalent of a 30 km drive in a small car (based on two separate visits of 90 minutes each). One major factor highlighted in the study is the use of single-use stainless steel - the second largest contributor to the carbon footprint after electricity use.^30^ While nickel titanium files are more common today, they come at a higher cost and may have a comparable or even larger carbon footprint than stainless steel files. Minimally invasive endodontic approaches offer a cost-effective, efficient and sustainable alternative, eliminating the need for multiple single-use instruments/several other disposable items and reducing treatment visits.

## How to perform vital pulp therapies: practical clinical guide

VPTs offer numerous benefits for patients, practitioners and dental public health. By outlining clear considerations, tools and step-by-step clinical protocols, we can demystify these procedures and equip practitioners with the knowledge needed to achieve optimal outcomes. This section will guide clinicians through the essential factors and techniques that enhance the success of VPTs, ensuring they are well-prepared for effective implementation.

### Pre-operative considerations

Assessing the tooth's sound tissue volume and restorability as a single unit but also considering the whole mouth treatment strategy to ensure such an approach is in the patient's best interest. Confirming a positive response to pulp tests, such as the cold test or electric pulp test, is suggested, along with an assessment of the periradicular tissues through determining any tenderness to percussion or palpation and assessing for swelling or presence of a sinus.

#### Pulpal diagnosis

Current diagnostic terms for the management of the pulp are commonly known as ‘reversible' or ‘irreversible' pulpitis and associated with the terms ‘symptomatic' or ‘asymptomatic' to describe the objective results from clinical tests and the reported symptoms from the patient.^31^ This terminology is contested as such terms may not reflect the histological status of the pulp and may not lead to adequate treatment.^32^ Alternative diagnostic terms have been proposed to simplify clinical decision-making, determining whether the pulp is ‘viable' or ‘non-viable'.^33^ Pulp diagnosis remains one of the greatest challenges in current research and clinical practice due to the limitations in accuracy from the current tools we use for diagnostic testing.^17^ A systematic review and meta-analysis investigating and comparing the accuracy of five dental pulp tests concluded that laser doppler flowmetry and pulse oximetry were the most accurate diagnostic methods. However, these tests are not commonly available in a clinical environment and are therefore not practical for clinical practice with the current technology available. Electric pulp testing showed a high sensitivity at detecting vital teeth but low sensitivity for non-vital teeth, whereas cold pulp testing had a moderate sensitivity for both vital and non-vital teeth.^18^ The challenge remains for detecting a threshold where pulp tissues can or cannot be saved as these tests correlate poorly with the histological status of the pulp tissues.^34^ Ongoing research is exploring the value and accuracy of biomarkers such as MMP-9 (matrix metalloproteinase-9) to predict pulp status locally from pulpal fluid with promising results.^35^ A new classification has been developed and proposed to better characterise the pulpal inflammation with corresponding symptoms and treatment option, thus promoting pulp vitality when appropriate (Table 3).^9^ Preliminary research using this classification to correlate clinical symptoms and treatment is showing promising results.^36^

**Table 3:** Comparison of classification systems for pulpal disease^9^^,^^31^

**American Association of Endodontists 2013**	**Wolters et al. 2017**
**Reversible pulpitis:** Discomfort is experienced when a stimulus such as cold or sweet is applied and goes away within a couple of seconds following the removal of the stimulus	**Initial pulpitis:** Heightened but not lengthened response to the cold test, not sensitive to percussion and no spontaneous pain
	**Mild pulpitis:** Heightened and lengthened reaction to cold, warmth and sweet stimuli that can last up to 20 s but then subsides, possibly percussion sensitive
**Symptomatic irreversible pulpitis:** Characteristics may include sharp pain upon thermal stimulus, lingering pain (often 30 seconds or longer after stimulus removal), spontaneity (unprovoked pain) and referred pain. Sometimes the pain may be accentuated by postural changes such as lying down or bending over and over-the-counter analgesics are typically ineffective	**Moderate pulpitis:** Clear symptoms, strong, heightened and prolonged reaction to cold, which can last for minutes, possibly percussion sensitive and spontaneous dull pain that can be more or less suppressed with pain medication
	**Severe pulpitis:** Severe spontaneous pain and clear pain reaction to warmth and cold stimuli, often, sharp to dull throbbing pain, patients have trouble sleeping because of the pain (gets worse when lying down). Tooth is very sensitive to touch and percussion
**Asymptomatic irreversible pulpitis:** Based on subjective and objective findings indicating that the vital inflamed pulp is incapable of healing and that root canal treatment is indicated. These cases have no clinical symptoms and usually respond normally to thermal testing but may have had trauma or deep caries that would likely result in exposure following removal.	

#### Radiographic considerations and periapical status

A good-quality intra-oral periapical radiograph will help determine the tooth's restorability and demonstrate the periapical status. Detection of periapical rarefaction does not mean the pulp is necrotic and VPTs are not viable.^37^^,^^38^

#### Remaining dentine thickness and carious lesion depth

The depth of the cavity in proximity of the dental pulp and the remaining dentine thickness (RDT) can inform clinicians on the prognosis and outcomes of VPTs. Following restoration, a RDT of 0.5 mm or or greater is necessary to avoid evidence of pulp injury.^39^ Carious lesion depth can be classified radiographically as ‘deep' or ‘extremely deep'; deep caries is defined as ‘radiographic evidence of caries reaching the inner quarter of dentine, but with a visible zone of dentine between the caries and the pulp, which carries a risk of pulp exposure during operative treatment' and extremely deep caries as ‘radiographic evidence of caries penetrating the entire thickness of the dentine with certain pulp exposure during operative treatment'.^6^ Extremely deep caries is associated with penetration of microorganisms into tertiary dentine and the pulp causing pulp inflammation, whereas deep caries is more associated with bacteria present only in primary dentine. Extremely deep caries is more likely to be associated with infection in the pulp and requiring more invasive VPTs, like partial or full pulpotomy.^40^

#### Pre-VPT restorative procedures

Depending on the clinical scenario and the remaining tooth structure, pre-endodontic build-up or gingival resection may be required to obtain an optimal marginal seal, ensuring that isolation and asepsis are achieved and help with improved outcomes.

### Clinical set up

#### Magnification

The ESE recommends the use of magnification during the management of exposed pulp. Although this recommendation isn't based on direct comparative trials, it is derived from the combined evidence from studies using and not using magnification, alongside the rationale that improved vision should aid in the effective management of exposed pulp tissue.^6^^,^^7^^,^^41^

#### Strict aseptic field

The use of a strict aseptic field with dental dam is considered essential for endodontic treatment.^7^ Indeed, bacterial contamination severely impairs pulpal healing and therefore the success of VPT.^5^ The ESE recommends the use of dental dam isolation for the management of deep carious lesions and any subsequent endodontic procedure required.^6^

### Important intra-operative factors influencing outcomes

#### Timing and size of pulp exposure

The management of pulp exposure depends on the size and timing of the exposure relative to the complete removal of dental carious tissue to reduce bacterial load. Larger exposures often have less predictable outcomes with direct pulp capping.^42^ However, using HCSC materials like Biodentine (Septodont, France) and mineral trioxide aggregate (MTA) for larger exposures may lead to better outcomes.^43^ In such cases, more invasive VPTs, such as partial or full pulpotomy, may be necessary to prevent pulp contamination and effectively remove superficially inflamed tissues.^6^

#### Pulpal bleeding and tissue removal

Bleeding is a key clinical indicator of a vital, inflamed pulp, and its presence during a VPT procedure confirms the viability of the tissue. Observing healthy bleeding is the most reliable sign when VPT is chosen as the preferred treatment. In contrast, grey discoloration of the pulp or the presence of pus typically indicates pulp necrosis and infection. However, superficial necrosis does not necessarily mean the entire pulp is compromised. In such cases, more invasive procedures like pulpotomy may still be successful, as deeper portions of the pulp could remain vital and capable of healing.^44^

**Fig. 4:**
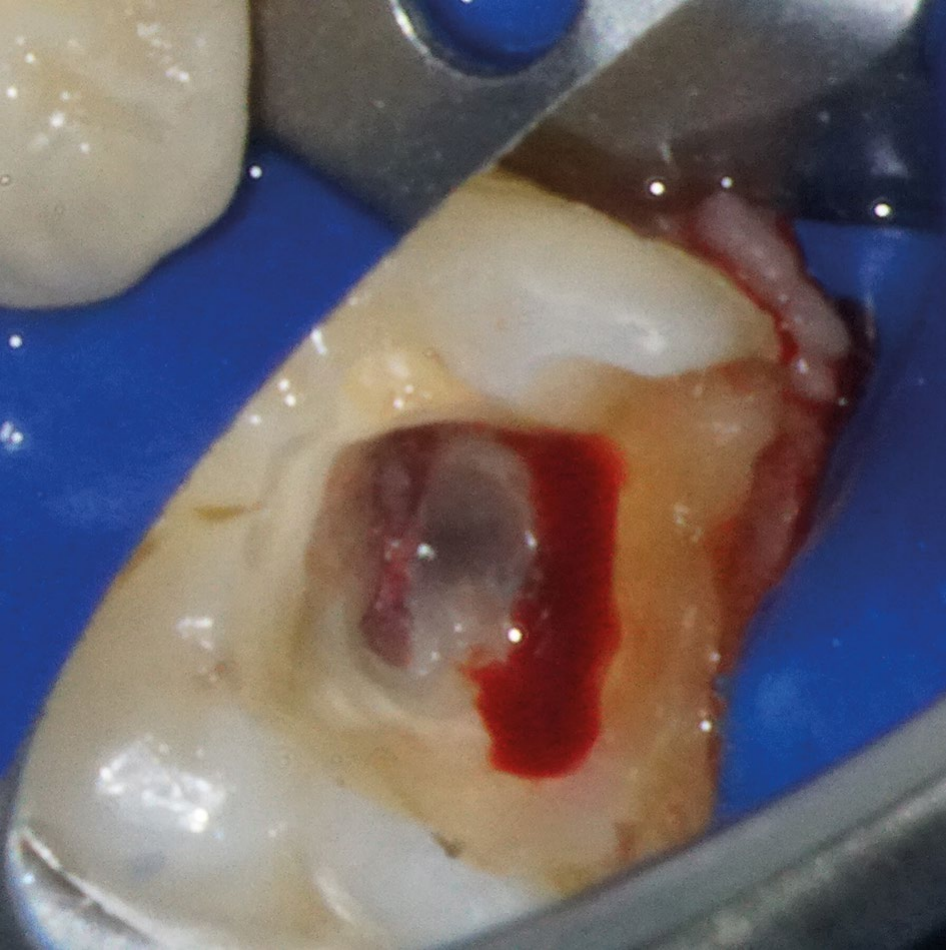
Attempting to obtaining haemostasis during pulpotomy of lower left first molar tooth. Successful control in mesial root but distal root still continues to bleed following first attempt with cotton wool pledget soaked in NaOCl application

A five-minute window is often recommended to achieve haemostasis, after which more invasive treatment may be necessary, as prolonged bleeding can indicate excessive inflammation, compromising pulp viability (Fig. 4).^6^ Although time to achieve haemostasis has not been shown to significantly affect the success rates of partial pulpotomy or full pulpotomy,^36^^,^^45^having a practical clinical cut-off is important, especially in emergency situations. Therefore, the five-minute window is regarded as both acceptable and realistic before considering more invasive interventions. Past this window, further invasive treatment, such as pulp tissue removal or pulpectomy, should be undertaken.

#### Pulpal lavage

NaOCl appears to be the solution of choice for pulp lavage in the event of VPT or RCT as it acts as a potent disinfectant and haemostatic agent. Its antimicrobial properties are beneficial and associated with increased healing of the pulp compared to saline solution, as demonstrated by Ballal *et al*.^35^ The study also demonstrated that NaOCl use was also associated with reduced immediate postoperative discomfort.

#### Presence of necrotic tissue in the pulp chamber

The presence of necrotic tissue in the pulp chamber requires careful assessment to determine whether any viable pulp tissue remains or if RCT is necessary. In cases of superficial necrosis or abscess, the removal of the affected tissue may reveal healthy pulp beneath, making a full pulpotomy a viable treatment option.^44^ In multi-rooted teeth, if partial tissue necrosis is identified, RCT may be performed in canals with necrotic pulp, while pulpotomy with HCSC may be considered for the remaining canals if they contain healthy pulp.^46^ However, if the pulp is deemed non-viable, a pulpectomy and complete RCT are recommended. The choice of treatment depends on the extent of necrosis and the operator's experience and skill.

#### Direct versus indirect pulp capping or partial/full pulpotomy

Determining the most favourable treatment strategy for managing pulpitis is multifactorial. Pre-operative clinical and radiographic investigations play a key role, as the diagnosis affects the level of VPT performed. However, intra-operative findings also influence decision-making.

A less invasive approach to caries management when treating pulpitis with initial or mild symptoms is more favourable and cost-effective.^47^^,^^48^ When the pulp is exposed with mild-to-moderate pulpitis symptoms, direct pulp capping (91% success at 12 months),^49^ partial pulpotomy (96% success at 12 months),^50^ and full pulpotomy (97% success at 12 months)^51^ using HCSC materials show high success rates. Additionally, direct pulp capping and pulpotomy procedures are generally more cost-effective than RCT.^52^

Pulpotomy procedures tend to be more predictable over a longer follow-up period. When managing severe or irreversible pulpitis, the success rate of partial pulpotomy decreases significantly,^50^ making full pulpotomy or RCT more predictable.^13^ Pulpotomy is less costly than RCT and the failure of initial therapies is also less costly for pulpotomy compared to RCT.^53^

### Materials leading to success

#### Hydraulic calcium silicate cements

**Fig. 5:**
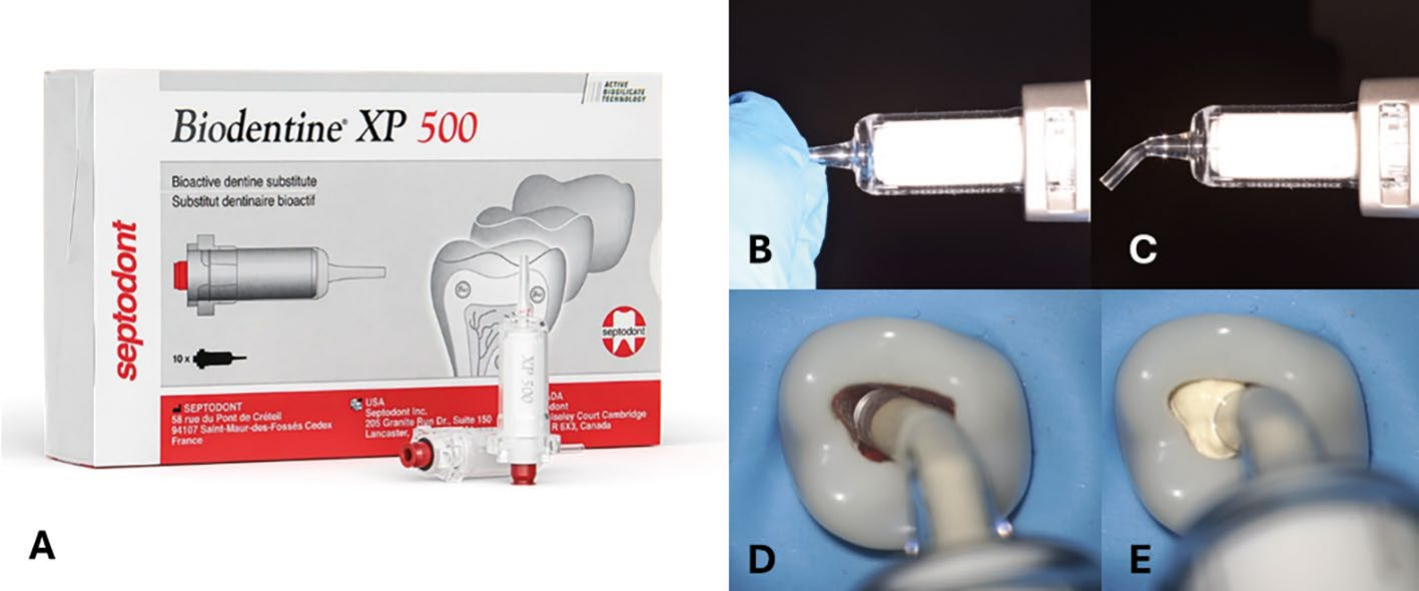
An example of a HCSC used for VPT. a) Biodentine XP which has an acceptable setting time and physical resilience to be exposed to the oral cavity once set. Image reproduced with permission from Septodont. b, c) Presented in a bespoke applicator. It can be modified to improve ergonomics. d, e) Directly applied to tooth undergoing pulpotomy

In the past 20 years, modern bioactive and biocompatible materials (HCSC ie ProRoot MTA; Biodentine, BC putty) have been developed with enhanced properties to stimulate healing and regeneration of pulpal tissues following VPT^54^^,^^55^(Fig. 5). Indeed, HCSCs are advantageous compared to calcium hydroxide [Ca(OH)_2_], with higher mechanical strength, lower solubility and better sealing of dentine.^56^^,^^57^Multiple studies investigating the biocompatibility and bioactivity of HCSC demonstrated their excellent sealing capacity and ability to promote hard tissue formation. Furthermore, materials such as MTA or Biodentine allow favourable proliferation of dental pulp cells^58^ and reactionary and reparative dentinogenesis, which is essential in VPTs with direct pulp capping.^59^ Although these studies were conducted *in vitro*, HCSCs have also demonstrated their superiority when compared to Ca(OH)_2_ as a dental pulp capping material in terms of dental pulp protection following an exposure.^60^

#### Calcium hydroxide

Ca(OH)_2_ has been traditionally used as an indirect and direct pulp capping material with a relative success up to 90% provided that a hermetic seal is obtained.^61^ However, longitudinal studies highlight a decrease in success over time, from 87.5% success at six months to one year, down to 72.9% success after more than three years.^62^ This may be due to the fact that Ca(OH)_2_ has limited properties (biological and mechanical) to create an adequate pulpal seal, which may lead to further bacterial contamination.^63^ Further research also revealed that Ca(OH)_2_ as a liner was unnecessary for the management of deep carious lesions both for primary and permanent teeth.^64^

### Which material should I use?

A practice-based randomised clinical trial comparing Ca(OH)_2_ and MTA for direct pulp capping highlighted 68.5% probability success with Ca(OH)_2_ and 80.3% probability of success after two years (success being defined as the tooth not requiring extraction or RCT).^65^ Despite a higher initial material and treatment cost, direct pulp capping with HCSC is both more effective and less costly compared to direct pulp capping with Ca(OH)_2._^48^^,^^66^This was also confirmed with a systematic review and meta-analysis comparing the efficacy of direct pulp capping of cariously exposed pulps in permanent teeth showing the superiority of HCSC over Ca(OH)_2_.^49^ Based on the mechanical, biological, cost-effectiveness and clinical superiority of HCSC evidenced previously, HCSCs are recommended for any VPT procedure.

### How to: step by step guide

**Fig. 6:**
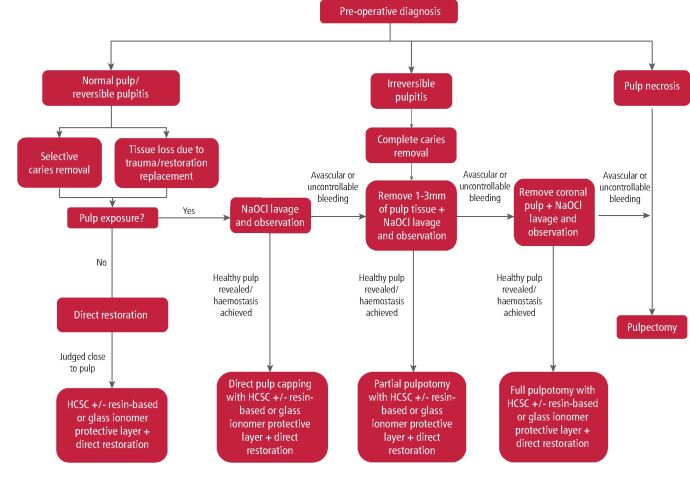
Flowchart for vital pulp therapies

Indirect pulp capping, direct pulp capping and partial/full pulpotomy are laid out in Table 4, Table 5 and Table 6, respectively.^67^^,^^68^^,^^69^ Figure 6 provides a flowchart for vital pulp therapies.

**Table 4:** Protocol for indirect pulp capping^7^^,^^67^

**Indications**	**Protocol**
Tooth free from symptoms or very mild pulpitis Deep cavity which is judged to be close to the pulp	Local anaesthesia Dental dam isolation + magnification/illumination if possible Disinfection of crown with NaOCl (1-5.25%) Initial cavity preparation with high speed and diamond bur with constant water cooling Management of carious tissue Cavity cleaned with NaOCl (1-5.25%) Placement of HCSC according to manufacturer's instructions towards the dental pulp tissue, keeping peripheral margins free Optional: depending on the material, consider placing (RMGIC/GIC/resin-based material) to separate the pulp capping material from the definitive restorative material Direct restoration
**Contra-indications**	
Unrestorable tooth Spontaneous pain

**Table 5:** Protocol for direct pulp capping^7^^,^^68^

**Indications**	**Protocol**
Tooth free from symptoms or very mild pulpitis Deep cavity with pulp exposure	Local anaesthesia Dental dam isolation + magnification/illumination if possible Disinfection of crown with NaOCl (1-5.25%) Initial cavity preparation with high speed and diamond bur with constant water cooling Complete caries removal (change cutting instrument for a new sterile one at close proximity to pulp) Cavity and exposure cleaned with NaOCl (1-5.25%) Pulp bleeding haemostasis achieved within 5 mins with gentle pressure with sterile cotton pellet soaked with NaOCl (if haemostasis not achieved or necrotic tissue present, follow pulpotomy protocol) Once pulp haemostasis achieved, placement of HCSC according to manufacturer's instructions towards the dental pulp tissue, keeping peripheral margins free Optional: Depending on the material, consider placing (RMGIC/GIC/resin-based material) to separate the pulp capping material from the definitive restorative material Direct restoration
**Contra-indications**	
Unrestorable tooth Spontaneous pain Severe prolonged bleeding or discharge of serous/purulent exudate from pulp Necrotic pulp tissue Periodontally damaged teeth Radiological features of intrapulpal mineralisation

**Table 6:** Protocol for pulpotomy^7^^,^^69^

**Indications**	**Protocol**
Extremely deep caries where pulp exposure is inevitable or is exposed during caries removal Mature tooth with deep carious lesion and symptomatic reversible/irreversible pulpitis Traumatically exposed pulp A multirooted tooth with localised severe periodontitis affecting one root which is planned to be resected	Local anaesthesia Dental dam isolation + magnification/illumination if possible Disinfection of crown with NaOCl (1-5.25%) Initial cavity preparation with high speed and diamond bur with constant water cooling Complete caries removal (change cutting instrument for a new sterile one at close proximity to pulp) Cavity cleaned with NaOCl (1-5.25%) Access to the pulp chamber with sterile high-speed bur. For full pulpotomy, the entirety of the roof of the pulp chamber needs to be removed Pulp tissue amputation with sterile high-speed bur or sharp sterile excavator Pulp bleeding haemostasis achieved within 5 mins with gentle pressure with sterile cotton pellet soaked with NaOCl (if haemostasis not achieved or necrotic tissue present, further pulp tissue removal or root canal treatment initiation) Once pulp haemostasis achieved, placement of HCSC according to manufacturer's instructions towards the dental pulp tissue, keeping peripheral margins free Optional: Depending on the material, consider placing (RMGIC/GIC/resin-based material) to separate the pulp capping material from the definitive restorative material Direct restoration
**Contra-indications**	
Unrestorable tooth Severe prolonged uncontrollable bleeding or discharge of serous/purulent exudate from pulp Necrotic pulp tissue Radiological features of intrapulpal mineralisation

### Postoperative considerations

#### Coronal seal and indirect restoration

The coronal seal is a critical factor in determining the success of both endodontic treatment and VPT.^70^^,^^71^Indeed, a reliable coronal seal is essential for restoring the health and function of the affected tooth. The same restorative principles used for endodontically treated teeth should be applied to those undergoing VPT.^10^ In cases where VPT-treated posterior teeth have lost one or more proximal walls, they should receive cuspal coverage through either a direct restoration or indirect restoration to ensure long-term structural integrity and function.

#### Postoperative pain and management

Postoperative pain is a common complication following VPT. A recent systematic review and meta-analysis comparing postoperative pain between single-visit RCT and VPT found that VPT generally results in less moderate-to-severe pain during the first 72 hours. Pulpotomy was associated with a significantly higher rate of no pain and a lower incidence of mild-to-moderate pain when compared to single-visit RCT. While direct pulp capping showed a very low incidence of moderate-to-severe postoperative pain, any mild-to-moderate discomfort that did occur tended to persist for a longer duration compared to other modalities.^25^ Another systematic review by Tomson *et al*. found no significant difference in postoperative pain after seven days between pulpotomy and RCT for the management of irreversible pulpitis.^13^ This was also confirmed with a recent randomised controlled clinical study comparing the short-term postoperative pain and impact on quality of life between pulpotomy and RCT. This showed that pulpotomy was as effective as RCT in reducing postoperative pain, improving quality of life and health-related quality of life.^72^

### Future considerations

#### Education

To successfully implement VPT, adequate education and training are essential at both undergraduate and postgraduate levels. Dental practitioners not only need to be familiar with the protocols for these therapies but also require access to and experience with HCSC and magnification tools, such as dental operating microscopes or magnification loupes. Chin *et al*. reported that dentists in Wales who participated in their survey often used setting Ca(OH)_2_ for vital pulp treatments instead of HCSC due to cost, lack of training, or limited exposure to these materials.^73^ Similarly, Edwards *et al*. found that the most common management for symptomatic irreversible pulpitis taught at the undergraduate level was pulpectomy or pulpotomy, followed by an antibiotic/corticosteroid dressing (66.7% and 56.2%, respectively).^74^ Dentists who considered full pulpotomy as a definitive treatment option were more likely to have a postgraduate qualification. These studies highlight several barriers faced by dental practitioners regarding the use of HCSC in VPT, including lack of training, concerns about the reliability of the treatment, limited access to these products, and issues related to cost and remuneration. Further qualitative research is needed to explore the teaching and training needs of undergraduate students and general dental practitioners to better inform the implementation of VPT.

#### Primary care translational research

Several publications on clinical research related to VPTs emphasise their advantages and benefits for patients, practitioners and dental public health. However, these studies are often conducted in secondary care settings, where treatments occur in highly controlled environments with few time or cost constraints. The authors acknowledge this discrepancy between secondary and primary care and recommend that future studies be conducted in primary care settings using more pragmatic approaches. This would help confirm whether such therapies can be successfully and sustainably implemented for most dental practitioners.^13^^,^^75^In the United Kingdom (UK), the National Institute for Health Research has sponsored two UK-wide, pragmatic, randomised controlled trials in primary care focused on VPTs (PIP and SCRIPT studies). One trial investigates the benefits and outcomes of selective caries removal compared to routine caries removal in permanent posterior teeth.^76^ The other explores the use of full pulpotomy for managing symptomatic irreversible pulpitis, comparing it with RCT for permanent posterior teeth.^77^ This type of translational research, which also incorporates health economic considerations, aims to accelerate the implementation of evidence-based dentistry in primary care.^78^

## Conclusion

VPTs are reliable and successful procedures that promote minimally invasive dentistry by preserving the essential functions of the dentine-pulp complex, maintaining natural tooth structure, and prolonging the restorative cycles of the tooth, which will ultimately improve patients' oral health-related quality of life. Sustainability is a key consideration in today's healthcare landscape, particularly within the value-based care framework of the UK's National Health Service. These therapies hold the potential for a positive long-term impact not only on patient outcomes, but also on clinician experiences and environmental sustainability. Results from multicentre, practice-based research will further inform the implementation and promotion of these treatments. Additionally, advancing education and providing opportunities for supplemental training with modern materials will support the wider adoption of VPTs by dental practitioners.
